# Adapting Chinese Qigong Mind-Body Exercise for Healthy Aging in Older Community-Dwelling Low-income Latino Adults: Pilot Feasibility Study

**DOI:** 10.2196/29188

**Published:** 2021-11-01

**Authors:** Zenong Yin, Cristina E Martinez, Shiyu Li, Martha Martinez, Kezhi Peng, William M Land, Sarah L Ullevig, Adelita Cantu, Sharon Falk, Arthur E Hernández, Catherine Ortega, Deborah Parra-Medina, Maureen J Simmonds

**Affiliations:** 1 Department of Public Health, The University of Texas at San Antonio San Antonio, TX United States; 2 School of Nursing, The University of Texas Health Science Center at San Antonio San Antonio, TX United States; 3 College of Kinesiology and Health, Guizhou University of Traditional Chinese Medicine Guiyang China; 4 Department of Kinesiology, The University of Texas at San Antonio San Antonio, TX United States; 5 College for Health, Community and Policy, The University of Texas at San Antonio San Antonio, TX United States; 6 Dreeben School of Education, University of the Incarnate Word San Antonio, TX United States; 7 Latino Research Institute, Latino Studies, The University of Texas at Austin Austin, TX United States

**Keywords:** mHealth, community-based participatory research, five animal play, wuqinxi

## Abstract

**Background:**

Research translating the evidence for the benefit of mind-body exercise in older Latinos with limited access to community-based healthy aging programs is sparse.

**Objective:**

This study aimed to evaluate the feasibility of Function Improvement Exercises for Older Sedentary Community-Dwelling Latino Residents (FITxOlder), a Community Health Worker (CHW)-led, mobile technology-facilitated Chinese Qigong mind-body exercise program for healthy aging and to explore its impact on physical and cognitive function and quality of life (QoL) in older community-dwelling low-income Latino adults.

**Methods:**

This study was designed as a Stage 1 feasibility study to develop and pilot-test FITxOlder. In Phase 1 (Stage 1A), a working group of seniors, CHWs, and senior center staff guided the adaptation of Chinese Qigong into a healthy aging program. In Phase 2 (Stage 1B), 49 older Latino adults participated in a 3-arm controlled study to test the feasibility and preliminary effect of CHW-led FITxOlder on physical and cognitive function and QoL measures over 16 weeks.

**Results:**

Although the COVID-19 pandemic disrupted the implementation of the study protocol, we found favorable results regarding participant recruitment, retention, and fidelity of implementation. Notable findings included an 89.3% participant retention, 79.4% of the participants completed at least 70% of the weekly exercise goal, and no report of adverse events. The effects on intervention outcome measures were modest.

**Conclusions:**

FITxOlder is feasible for promoting healthy aging in older Latino adults; future research needs to compare its feasibility with other low-impact exercise programs for healthy aging using a randomized controlled trial.

**Trial Registration:**

ClinicalTrials.gov NCT04284137; https://clinicaltrials.gov/ct2/show/NCT04284137

## Introduction

Latinos/Hispanics aged 65 years and older are the fastest-growing racial/ethnic group and the most overrepresented low socioeconomic status population in the United States [1]. Compared to non-Hispanic Whites, older Hispanics have lower levels of physical and mental health, lower quality of life (QoL), and higher levels of impairment when performing activities of daily living (ADL) and other instrumental activities [2]. Aging is also associated with deteriorating health conditions, and 80.1% of Americans aged 65 years and older have 2 or more chronic conditions that further exacerbate ADL impairment and social and cognitive functions [3]. In addition, being a minority, female, and uninsured increases the risk of having multiple chronic conditions [4,5]. For example, Latino females (46.4%) and Black adults (46.7%) aged ≥65 years had the highest prevalence of having ≥4 chronic conditions among the Medicare beneficiaries. Although regular participation in physical activity (PA) is an effective approach to reducing the risk of chronic conditions and promoting healthy aging and independent living [6], the majority of older Americans do not meet the recommended level of PA [1], and over a quarter of older Latino adults do not participate in adequate PA, including leisure-time PA [7]. Ongoing efforts have considered the health conditions, previous PA habits and experiences, cultural preferences, social support, convenience, accessibility, and use of mobile health (mHealth) technologies to promote PA in community-dwelling older adults [8,9], with mixed results [10].

There is increased interest among Americans in mind-body exercises originating from non-Western regions and cultures, and they have been deemed safe, beneficial, and cost-effective therapeutic forms of complementary and alternative medicine (CAM) [11,12]. Chinese Qigong, Tai Chi, and Yoga are commonly known mind-body exercises with roots in ancient Asian culture [13]. Under the broad umbrella of Dao Yin, Qigong and Tai Chi are medical treatment and health preservation practices in traditional Chinese medicine [14], characterized by (1) slow body movements that symbolize the movement or posture of animate objects or natural events with low to moderate physical exertion, (2) coordinated breathing that is consciously or automatically (expertly) controlled, and (3) a state of mental quieting (ie, a meditative state) that is achieved by calming the mind or disassociating from disruptive thoughts [15,16]. Evidence from randomized controlled trials (RCTs) indicates that Qigong and Tai Chi have clinically significant effects on physical and cognitive functions [14,17-19], QoL [20], immunomodulatory or inflammatory responses [21,22], and chronic physical and mental health conditions and disorders [18,19,23] in older adults and populations with chronic conditions. Given the repetitive nature and lower level of physical exertion, Qigong exercises tend to be easier to learn and more appealing to older adults and individuals with chronic health conditions than Tai Chi and Yoga [17,20,24-26]. Qigong can be delivered at a low cost with minimum safety concerns in community locations and homes and thus, are affordable and sustainable [25-27].

While Qigong appears to be an appealing form of PA for older adults, to date, no research exists on the effectiveness of Qigong among low-income Latino adults that have experienced disparities in accessing community-based healthy aging programs [28,29]. Separately, research is limited on evidence-based interventions (EBIs) involving mHealth among older minority adults [30]. Furthermore, indiscriminate cross-cultural adaptation of EBIs may reduce effectiveness and impede sustainability [31,32].

To address this knowledge gap, we evaluated the feasibility of a Qigong healthy aging program in community-dwelling low-income Latino adults with chronic health conditions following the National Institutes of Health’s (NIH) Stage Model [33]. As such, the study's primary aim was to assess the feasibility of an adapted Qigong program led by community health workers (CHWs) and facilitated by mHealth. A second aim was to explore the effect of the Qigong program on healthy aging-related outcomes. The study results will be used to plan a large RCT for testing the efficacy of Qigong in promoting healthy aging.

## Methods

### Study Design

We conducted a 3-arm study to assess the feasibility of a Qigong program for healthy aging, called Function Improvement Exercises for Older Sedentary Community-Dwelling Latino Residents (*FITxOlder*) among low-income Latino participants in the inner-city area of San Antonio, Texas. Designed as an NIH Stage 1 feasibility study [34], the research consisted of 2 phases: Phase 1, a formative study for intervention development (Stage 1A) that was used to develop and adapt *FITxOlder* and create training materials to facilitate the study implementation, and Phase 2, pilot testing of *FITxOlder* (Stage 1B) to evaluate whether the intervention was feasible to implement with and acceptable to the intended study population. The changes in the outcome measures were used to estimate the effect size for designing the healthy aging RCT [34]. The Institutional Review Board of the University of Texas San Antonio approved the study protocol.

### Study Setting and Recruitment

The study sites were senior centers that served predominantly Latino, low-income adults who were participants of the congregate meals program. The Phase 1 formative study was conducted at a senior center where all program participants self-identified as Latino, and participation in the working group was voluntary. Participants for the Phase 2 feasibility testing of the pilot intervention were recruited through flyers distributed at 3 senior centers. Interested individuals provided their contact information to senior center staff, then research staff conducted eligibility screening based on established criteria—age ≥60 years, ability to exercise in a standing position, ownership of a cell phone or living with someone with a cell phone, agreement not to participate in other concurrent health programs, and willingness to complete the 3-month study. We did not screen for chronic conditions since over 80% of older adults had ≥3 chronic conditions in a similar congregate meals program in San Antonio [35]. Eligible individuals completed a consent form and signed up for baseline assessment. All recruitment and consent materials were available in Spanish and English. The recruitment goal was 15-20 participants at each of the 3 sites with ≥25 program participants. The participants received US $30 for completing the baseline assessment and US $40 after completing the posttest.

### Description of the Study and Intervention

#### Phase 1-Formative Study: Intervention Development

We conducted the formative study guided by the principles of community-based participatory research (CBPR) [36] and the framework for the design and adaptation of EBIs in health disparity populations [37]. The Phase 1 goal was to develop a culturally tailored exercise program for healthy aging that would incorporate low-cost mind-body exercises and address PA barriers and facilitators to improve physical and cognitive function and QoL among older low-income Latino adults. A concurrent goal was to develop a healthy aging instructor training program that could be scaled to prepare community-based program instructors for the pilot study in Phase 2.

The initiation of Phase 1 involved convening of a multidisciplinary research team (ie, physical therapy, community nursing, community health, nutrition, and exercise science or psychology) and conducting a state-of-the-art literature review to critically assess current and emerging evidence and identify issues and needs of future research in PA intervention targeting physical and cognitive function and QoL in older adults, focusing on mind-body exercises (see Table S1 Multimedia Appendix 1 for a summary of results). Based on this review, we decided to move forward with 2 forms of Qigong, Five Animal Play or Five Animal Frolics (*Wu Qin Xi* in Chinese) [38] and Eight Pieces of Brocade or Eight-Section Brocade (*Ba Duan Jin* in Chinese) [39]. Five Animal Play consists of 5 sets of choreographed movement routines that symbolize “the courage and robustness of the tiger; serenity and poise of the deer; the steadiness and solidity of the bear; the nimbleness and dexterity of the monkey; and the swiftness and grace of the bird” [40]. The routines combine stretching, balancing, weight-bearing, and eye-hand coordination movements blended with controlled breathing and mental immersion in the “mindset” of each specific animal. Eight Pieces of Brocade consists of 8 sets of primarily stationary movements designed to stretch and strengthen different body parts in conjunction with coordinated breathing and mind-focus.

The subsequent 3-month period involved engaging a 12-member working group of seniors, CHWs, and senior center staff in weekly meetings to co-design *FITxOlder* and provide guidance and feedback to the pilot study protocol. These weekly meetings were held at the senior center and scheduled at the end of the day. English-Spanish bilingual research staff facilitated the meetings. Participating seniors received US $5 for attending each session. At the meetings, research staff presented the goals of the study and discussed the principles of CBPR. Other topics included the historical and cultural background of traditional Chinese medicine and Qigong exercises, symbolism and metaphors in Chinese culture and Qigong exercises, different versions of Five Animal Play and Eight Pieces of Brocade, and research evidence related to the benefits of Qigong exercises. The working group provided input on their understanding of the information presented and experiences of learning and practicing Qigong and their Latino perspective on a traditional Chinese cultural practice. Group discussions compared Qigong to Latino culture and symbolisms, and the participants offered specific suggestions on how to modify Qigong to accommodate their personal and cultural preferences and needs (eg, symbolization of animals, background music, instructor qualifications, safety concerns, challenges in learning complex routines) from a perspective of older Latino adults living in a low-income community. Given the heterogeneity of Latino culture [41], the working group could not offer clear guidance to tailor the symbolism and metaphors in the Qigong culturally. Therefore, we opted to focus on adapting the program delivery process and movement routines to address feedback regarding movement complexity, level of exertion and balance, and safety.

As a result of the CBPR meetings, we decided not to introduce “Qi” or “flow of energy,” a concept in traditional Chinese medicine, in the Phase 2 pilot intervention. Although Five Animal Play and Eight Pieces of Brocade*s* are similar mind-body exercises, the former is easier to learn and quite suitable for older adults, and thus, was chosen as the exercise for the study. Working group members also provided input on strategies (eg, using a video model to facilitate independent exercise at home) to promote acceptance, adoption, and sustainability of Qigong for home practice. Finally, the working group provided feedback on the study protocol regarding recruitment, retention, assessment, and safety. During this period, seniors at the senior center, including the working group members, voluntarily participated in learning and practicing the official version of Five Animal Play (OfficialFAP) and Eight Pieces of Brocades every week facilitated by in-person instruction of research staff and CHWs using videos.

Key elements of *FITxOlder* included a video-guided mind-body exercise (ie, Five Animal Play), biweekly group practice sessions, program delivery and social support by trained CHWs, a goal-based home practice program, and facilitation by mHealth technologies to enhance participation and engagement in the *FITxOlder* program. Following social-cognitive theory, *FITxOlder* was designed to increase participants’ efficacy to learn and practice Five Animal Play using vicarious experience, goal setting, reinforcement, role modeling, social support, and self-regulation [42]. Expected benefits of the intervention program were improved physical and cognitive function and QoL, which constituted the foundation of healthy aging and independent living [29].

The planned progression of a 12-week *FITxOlder* was guided by the 3 core principles of harmonization (Tiao Shen, Tiao Xi, and Tiao Xin) in Qigong practice [38,43]. Weeks 1-4 focused on harmonizing or tuning one’s posture and body movements to symbolize the tiger, bear, monkey, bird, and deer (Tiao Shen). The CHW introduced the Five Animal Play as a form of Chinese body-mind exercise to improve physical and cognitive function. Participants learned to perform the Five Animal Play following a demonstration by CHWs and a display of a study-produced video on a large screen. Weeks 5-8 emphasized harmonizing or tuning the breath with movement routines (Tiao Xi). Participants learned to blend inhaling air with outward or extension movement and exhaling air with inward or flexion movement. Weeks 9-12 focused on harmonizing or tuning one’s mind to reach a state of mental quieting and avoiding disruptive thought by uniting body, breath, and mind into “one” (Tiao Xin). This practice involved mentally immersing oneself into the animal being performed with blended breathing to reach a meditative state [44]. Participants’ exercise goals were (1) to attend 2 weekly group sessions and (2) to practice Five Animal Play at home following a 26-minute video, at least once a week in weeks 1-4, 2 times a week in weeks 5-8, and 3 times a week in weeks 9-12.

OfficialFAP [39] begins with an abdominal breathing routine to focus the mind on the body, followed by 5 sets of routines symbolizing 5 animals (2 subroutines per animal). Each set starts and ends with slow controlled breathing. The exercise finishes with an abdominal breathing routine to refocus the mind to an awaking state. Five Animal Play is usually practiced following a model who leads the exercise following a video or audio recording with traditional calming Chinese music in the background. The entire exercise takes approximately 13 minutes to complete [38]. To examine its feasibility and acceptability, we produced 2 versions of English-guided videos demonstrating Five Animal Play based on the working group's suggestions. The videos were 26 minutes total in length, repeating the 13-minute exercise once. Participants could stop the video between the 2 sets to take a break as desired. The first version was the OfficialFAP video, in which Chinese models performed the exercise following translated English voice cues. The second version involved trained CHWs and research staff performing the modified version of Five Animal Play (ModifiedFAP) accompanied by English language action cues. The modifications were made to reduce complexity in 2 subroutines (monkey subroutine 2 and deer subroutine 2) and difficulty of movements requiring one-leg support (weight-bearing) and a high degree of balance. This was done to increase a sense of mastery, reduce the level of physical exertion, and address concerns for safety. The voice cues in both videos were modified to provide detailed movement guidance, emphasize the timing of inhaling and exhaling breath, and offer mental images of the movement (eg, “pick the fruit like a monkey” and “raise arms like a bird”).

The final Phase 1 activity involved developing a CHW training program and training 2 English-Spanish bilingual CHWs to deliver the pilot intervention in Phase 2 of the study. The CHW training included (1) the history, cultural background, and health benefits of Qigong, (2) study protocol and human subject protection, (3) instruction and safety in leading exercises with older adults, (4) learning of Five Animal Play, and (5) assignment of home practice. The CHWs received a 2-hour in-person instruction for performing Five Animal Play by a study team member (KP) who had training in traditional Chinese medicine and practiced and taught Qigong exercises to medical students and inpatient and outpatient populations in China over 15 years. The CHWs also practiced the Five Animal Play at home using the video daily for 2 weeks. One CHW was trained to deliver the Five Animal Play without modification, and the other was trained to deliver the ModifiedFAP.

#### Phase 2-Feasibility Testing of the Pilot Intervention

Phase 2 pilot-tested the feasibility of a 12-week *FITxOlder* exercise program in a 3-arm controlled trial at 3 community centers. Arm 1 participants received *FITxOlder* with in-person instruction and video-guided practice following the OfficialFAP without modifying the movements. At another center, arm 2 participants received *FITxOlder* with in-person instruction and video-guided practice using the ModifiedFAP. The *FITxOlder* delivery (ie, duration, exercise goals, incentive schedule, and support) was identical in both arms 1 and 2. The mHealth component of the intervention included using an Android tablet for playing the Five Animal Play video, text reminders, and telephone calls for support. Arm 3 was a placebo control treatment with a healthy aging program at the third center. Treatment assignment was based on travel distance for each CHW and research staff, with the furthest center assigned to control treatment. All participants received an orientation to the study as part of the baseline assessment, which presented the goals, expectations, safety issues, and study schedule. Instruction in all treatment arms was offered in English since the majority of the participants spoke English. The bilingual CHWs used Spanish to communicate with individual participants who had limited English proficiency or preferred Spanish.

The CHW led the biweekly 60-minute sessions comprising of (1) greet-and-meet, attendance check, and review of activities from the previous week, (2) warm-up, (3) performing Five Animal Play led by CHW while playing the study-produced Five Animal Play video on a big-screen monitor, (4) teaching Five Animal Play with a part-whole method, (5) performing Five Animal Play following Five Animal Play video while the CHW circulated the room and worked with individual participants, (7) cool-down, and (8) closing activities (ie, assigning home exercises for the week, addressing study-related problems, and completing the exercise log and exercise feeling survey). For safety, participants were instructed to perform the exercise to the best of their ability and avoid pain, exhaustion, or unpleasant feelings. The participants could use an assistive device (eg, a walker) for support or sit in a chair at their discretion. Participants were encouraged to ask questions and share their experiences and problem-solving strategies with each other. Each CHW was responsible for developing session lesson plans and documenting the process. Table S2 in Multimedia Appendix 1 presents the layout of the group sessions.

Each intervention participant received a 10-inch Android tablet to play the study-produced Five Animal Play video at home. During the first week, participants were taught how to use the tablets. They also received a study handbook that included exercise goals, weekly exercise logs, adverse event logs, incentive schedule, exercise safety and motivation tips, and study contact information. There were specific instructions on recording their home practice (eg, number of times and problems or issues) in the weekly exercise log.

To increase compliance with home practice expectations, each participant received a text reminder twice per week in their preferred language (English or Spanish). Text messages were sent automatically using Remind, a widely used cloud communication platform to manage and send text messages to a large audience. Participants who missed an on-site session received a phone call from the CHW, encouraging them to make up the missed session at home. As an incentive, a small trinket worth US $2-$7 was offered upon reaching 70% of the exercise goal (ie, number of biweekly sessions and home practices) during each 4-week period.

Program participants enrolled at the placebo control center received a 12-week evidence-based healthy aging program based on Aging Mastery by the National Council on Aging [45] delivered by the CHWs. Each participant received a workbook and attended weekly instructor-led sessions. Healthy-aging content included exercise, nutrition, finances, advance care planning, community engagement, and healthy relationships. Upon program completion, each participant received a program t-shirt and a US $25 gift card.

Due to the COVID-19 pandemic lockdown, all study sites were closed upon completion of week 4 of the 12-week program on March 15, 2020. As a result of stay-at-home orders and federal COVID-19 guidelines on social distancing, we changed the delivery of the intervention with weekly individual phone calls with participants in the 3 treatment groups aimed at following the original program protocol as closely as possible. Following the original 12-week plan, the CHW called their assigned participants weekly to continue the progression of developing harmonization of breathing (weeks 5-8) and mind (weeks 9-12). Each week, the CHW focused on 1 or 2 routines and answered questions related to the practice of Five Animal Play. The study timeline was extended an additional 4 weeks to offer further reinforcement and support and increase participants’ confidence and proficiency in practicing Five Animal Play (weeks 13-16). During these weekly calls, CHWs recorded intervention group participant progress and frequency of home practice. CHWs also called control group participants weekly to review the content of the Aging Mastery curriculum and to answer any questions about the program. During calls with all participants, the CHWs also inquired about each participant’s well-being and offered pandemic-related information and support per local, state, and federal guidelines. CHWs made up to 3 calls per week to reach each participant. There was no program activity provided for control participants during weeks 13-16. In addition, due to COVID-19 restrictions, we could not provide the Five Animal Play instruction for control participants after the completion of the study. Textbox 1 shows the objectives and activities for a 16-week revised study protocol of *FITxOlder*.

**FITxOlder Intervention timeline and intervention activities protocol.**

**Weeks 1 to 4**

- Two 60-minute group sessions led by a CHW each week
- Introduction of Five Animal Play to participants and learning choreography of the movement routines
- Practice of Five Animal Play at least one time at home following a video on a tablet
- Weekly text reminder to perform the exercise

**Weeks 5 to 8**

- Practice of Five Animal Play at least 4 times at home each week following a video on a tablet
- Instruction on blending movements and breathing and support by phone call
- Weekly text reminder to perform the exercise
- Weekly call to the participants by CHW to continue the instruction on integrating movements with breathing and provide social support

**Weeks 9 to 12**

- Practice of Five Animal Play at least 5 times at home each week following a video on a tablet
- Instruction on blending movements, breathing, and mind into “one” and support by phone call
- Weekly text reminder to perform the exercise
- Weekly call to the participants by CHW to continue the instruction on integrating movements with breathing and mental focus and provide social support

**Week 13 to 16**

- Practice of Five Animal Play at least 5 times at home each week following a video on a tablet
- Reviewing, reinforcement, and support by phone call
- Weekly text reminder to perform the exercise
- Weekly call to the participants by CHW to review Five Animal Play and provide social support

Note: All study sites were closed at the end of week 4 due to the COVID-19 pandemic.

### Study Measures

#### Primary Aim Measures

Measures of program feasibility included participant recruitment and retention, the fidelity of implementation, and reports of adverse events. Specifically, we defined recruitment success as a minimum of 60% (15 or 25+) enrollment at each study site and 80% retention. The participation target was at least 70% attendance at the biweekly sessions and 70% completion of weekly exercise goals. We also expected to successfully contact at least 50% of the participants through weekly telephone calls. CHWs tracked exercise session attendance and weekly phone calls. Each week participants recorded how many times they practiced the Five Animal Play on their exercise logs. The CHWs collected the practice information at a subsequent meeting or phone call. The CHWs also gathered pertinent information about participants’ health conditions and adverse events, as well as explanations for not attending the group session or not reaching the weekly exercise goals. Participants also completed a survey to provide demographics, health history, past experience using tablets, and willingness to participate in future studies.

To assess the reactivity to practicing 2 different versions of Five Animal Play, all participants completed the exercise-induced feeling inventory (EFI) [46] after completing the exercise. The 4 EFI subscales captured feelings of revitalization, tranquility, positive engagement, and physical exhaustion on a 5-point scale from 0 to 4, where 0 stands for “do not feel at all” and 4 stands for “feel very strongly.” In terms of formative assessment, the CHWs took notes documenting the participants’ progress in learning the routines in their weekly logs, and the research team staff also observed the biweekly sessions.

The original plan was to conduct a structured assessment of participant proficiency in performing the Five Animal Play once every 4 weeks. However, due to the COVID-19 epidemic, we were not able to conduct this participant proficiency assessment. At the conclusion of the study, participants in both intervention and control groups completed the client satisfaction questionnaire-8 (CSQ-8 )[47] to assess their satisfaction with the *FITxOlder* program. CSQ-8 scores range from 8 to 32, with higher scores indicating higher levels of satisfaction.

#### Exploratory Aim Measures

*FITxOlder* Exploratory aim measures included assessment of physical and cognitive function, QoL, chronic pain, and mindfulness to evaluate the effect of *FITxOlder* on healthy aging (see Table 1). These measures are outcome measures associated with healthy aging and risk for chronic conditions [29,48]. Trained nursing students, research assistants, and CHWs collected the assessment data at baseline and posttest. All questionnaires and surveys were offered in English or Spanish, depending on participant preference. Over the course of 2 weeks, 3 separate attempts were made to reach a participant for the posttest assessments. Given the COVID-19 pandemic restrictions, we modified the data collection protocol and collected data by telephone rather than in person. However, this change precluded our ability to conduct physical function tests. Therefore, participants completed a physical function self-assessment using the basic ADL and intermediate ADL subscale of the functional status questionnaire (FSQ) [49]. We were unable to collect posttest data on the symbol-digits modalities test [50] via telephone. We excluded the data from the Five Facet Mindfulness Questionnaire-Short Form since participants had difficulty comprehending the questions about mindfulness.

**Table 1:** The description of outcome measures and data collection timepoint.

Outcome measure	Baseline	Posttest
Physical functions: Research staff tested the participants with a battery of physical function tests [51], including the five times sit to stand test, 50-foot fast walk, 6-minute walk for distance, and forward lean reach, and measured the participant’s biometrics (height, weight, and blood pressure).	Yes	No
Self-assessment of physical functions: Participants completed the basic activities of daily living (eg, eating and dressing) subscale and intermediate activities of daily living (eg, light exercise, using public transportation, and housework) subscale of the functional status questionnaire.	No	Yes
Cognitive function: Participants completed the symbol-digits modalities test with a time limit of 90 seconds [50].	Yes	No
Quality of life: Participants completed the 12-item health-related quality of life (short-form 12 health survey) to generate a physical component score and a mental component score of quality of life [52].	Yes	Yes
Chronic Pain: Participants completed the 9-item brief pain inventory to measure perceived pain in two domains: pain severity and pain interference with life [53].	Yes	Yes
Mindfulness: Participants completed the 15-item five facet mindfulness questionnaire-short form to measure the deliberate and nonjudgmental attentiveness to present-moment experiences [54].	Yes	No

### Data Analysis

Given the study goal and focus of the *FITxOlder* program, the analysis focused on participants who self-identified as Latino (49/56, 87.5%). For the data analysis, we combined data from both the OfficialFAP and ModifiedFAP groups, given there were no discernable group differences across all the measures (ie, retention, attendance in weekly sessions, compliance with practice goal, reactivity to Five Animal Play during the first 4 weeks, and program satisfaction).

We summarized participant demographic data and other study-related characteristics using means and standard deviations for continuous and categorical variables tabulated as percentages. Depending on the normality of data distribution for continuous variables, we conducted a paired *t* test or Mann-Whitney U test to assess group differences. Contingency tests were used for categorical variables. We employed descriptive statistics to assess feasibility, retention, and implementation fidelity. General linear modeling was employed to explore the effects of the intervention on outcome variables at posttest, adjusting for baseline and demographic variables. Given that physical functioning was an important study outcome that was not assessed during the posttest, we created a proxy physical function index using a battery of physical function tests. Thus, we adjusted the index of physical function from the baseline to explore the effect of the intervention on the basic ADL and intermediate ADL scores of the FSQ posttest. As previously noted, we excluded the symbol-digits modalities test and Five Facet Mindfulness Questionnaire-Short Form scores in data analysis. Only significant covariate variables were retained in the model. We reported results as the adjusted difference between groups at posttest with CIs and *P* values. We calculated effect sizes (Cohen *d*) for the effect of the intervention on the study outcome variables. All analyses were conducted using SPSS software (version 27; IBM).

## Results

### Overview

Out of 64 study participants who met the study eligibility criteria, 87.5% (56/64; 49/64, 87% Latino; 4/56, 7% Black; and 3/56, 6% other racial/ethnic groups) completed the baseline assessment and were assigned to a treatment group. This included 17 in the OfficialFAP (15 Latinos), 19 in the ModifiedFAP (19 Latinos), and 20 in the control group (15 Latinos). Fifty baseline participants (50/56, 89%; 44/50, 88% Latino) completed the posttest assessment. Among those who did not complete the study, reasons included loss of contact (4/6, 66%), relocation (1/6, 22%), and loss of interest (1/6, 22%). We were able to reach 38% (21/56), 21% (12/56), and 31% (17/56) of the participants on the first, second, and third data collection calls. There were no differences in demographic characteristics between the completers and noncompleters of the study.

There were no differences between 49 Latino participants’ demographic characteristics (Table 2). The average age of intervention participants was 74.9 years (SD 6.3), compared to 73.9 years (SD 8.3) among controls. The majority were female and unmarried and reported no use of an assistive device. At study onset, 42.6% (21/49) of the intervention participants reported knowing how to use a tablet computer. Of particular note, nearly all participants expressed willingness to participate in future studies with the study team. Table S3 Multimedia Appendix 1 reports participant health information. The majority had been diagnosed with high blood pressure (28/49, 57%) or high cholesterol (30/49, 61%) or reported physical pain of lower extremity (28/49, 57%). Nearly half (24/49, 49%) reported 2 or more chronic health conditions (ie, high blood pressure, heart trouble, increased anxiety or depression, stomach problem, and vision or hearing problem).

**Table 2:** Demographic and study-related characteristics of study participants at baseline and posttest.^a^

Variable	Intervention (n=34)	Control (n=15)
Age (years), mean (SD)	74.9 (6.3)	73.9 (8.3)
Sex (female), n (%)	30 (88.2)	12 (80.0)
Education (<high school), n (%)	9 (26.5)	9 (60.0)
Currently working (yes), n (%)	2 (5.9)	2 (13.3)
Marital status (married), n (%)	7 (20.6)	3 (20.0)
Living with someone (yes), n (%)	10 (29.4)	7 (46.7)
Using an assistant device (yes), n (%)	7(14.2)	1 (7.7)
Language use (Spanish), n (%)	8 (23.5)	8 (53.3)
Knowing how to use a tablet computer (yes), n (%)	24 (42.6)	N/A^b^
Expressing willingness to participate in future studies (yes), n (%)	29 (97.0)	15(100.0)

^a^One-way *F* test was used to test the difference between intervention and control groups.

^b^N/A: not applicable.

### Attendance and Compliance of Weekly Exercise Goal

Average attendance at the 8 biweekly sessions was 80.1%. Reported practice of Five Animal Play at home ranged from 3 to 4 times a week. The mean percentage of participants who completed at least 70% of the weekly exercise goal was 79.4% (median 100%), ranging from 60.7% to 82.8% (Table 3). The mean percentage of participants who completed the weekly calls with CHW was 61.3% ranging from 56% (median 2 calls) to 69% (median 3 calls) every four weeks from week 5 to week 16.

**Table 3:** Class attendance, the number of times the exercise was practiced at home, and adherence to the monthly exercise goal.

Activity	Weeks 1-4, n=34	Month 5-8, n=28	Month 9-12, n=26	Month 13-16, n=29
Number of times attended the biweekly group sessions, mean (SD); median	6.41 (1.9); 7	N/A^a^	N/A	N/A
Number of times practiced Five Animal Play at home^b^, mean (SD); median	3.0 (1.2); 3	3.1 (1.4); 3	4.0 (1.8); 4	3.4 (1.7); 3
Percent of participants reached 70% of the weekly exercise goal^c^, mean (SD); median	79.40% (40.0); 100%	60.70% (49.7); 100%	73.10% (46.5) 100%	82.80% (38.4); 100%
Number of times called by the Community Health Worker, mean (SD); median	N/A	2.74 (1.52); 3	2.24 (1.54); 2	2.38 (1.42); 2.5

^a^N/A: not applicable.

^b^For month 1, the total number of times participants attended the class and the number of times the exercise was practiced at home.

^c^Monthly exercise goal is to complete at least 70% of expected times to practice the exercise for each month.

### Reactivity to Five Animal Play

**Figure 1:**
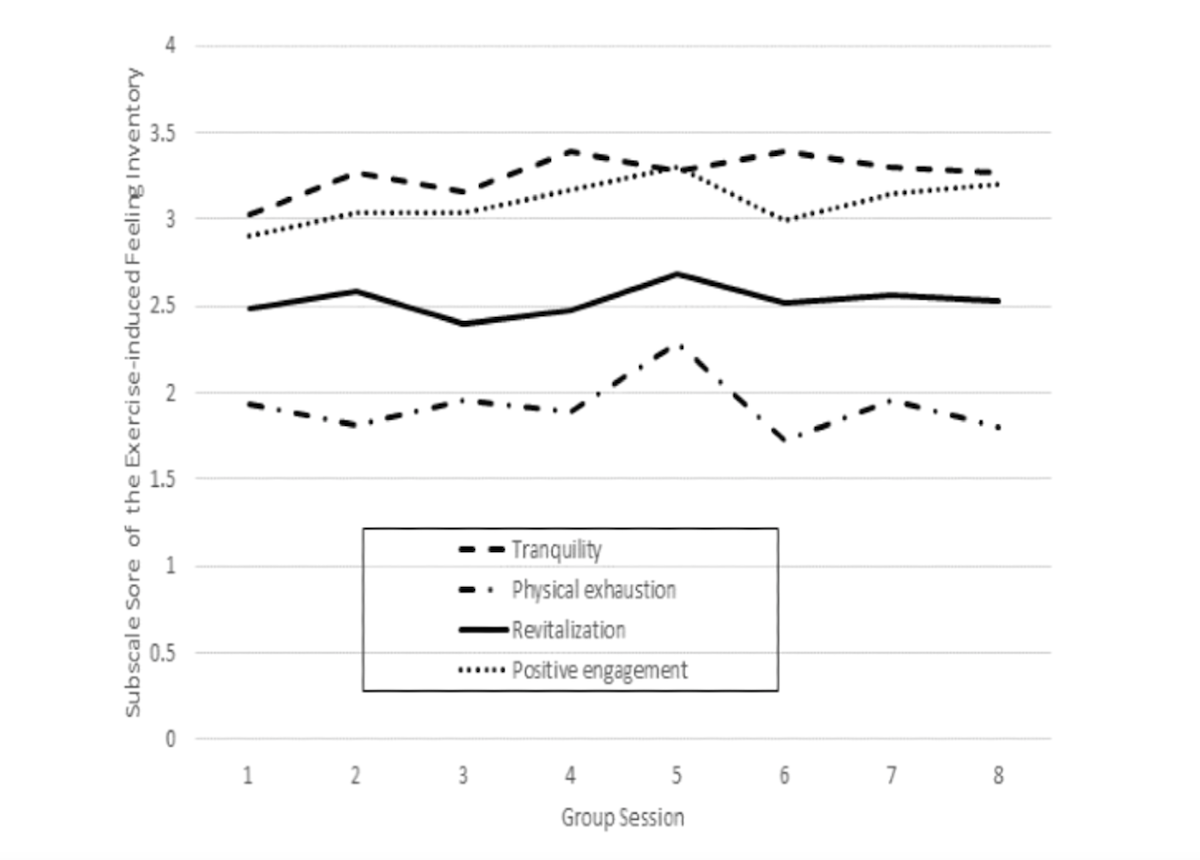
Subscale scores of the Exercise-induced Feeling Inventory from Group Sessions 1 to 8.

Participant responses related to exercise-induced feelings over the 8 biweekly sessions are presented in Figure 1. The physical exhaustion scores were in the middle range, and revitalization scores were slightly above the mean. The tranquility and positive engagement scores were in the upper range and showed a gradual increase over the 8 sessions.

#### Satisfaction With FITxOlder

Participants in the intervention (mean 30.8, SD 1.7) and control group (mean 30.6, SD 2.1) reported high satisfaction levels with the services and program offered.

#### Report of Adverse Event

No study-related adverse events were reported by participants based on documentation of the study team. We did not document the level of use of assistive devices during the biweekly sessions and home practices among the participants who reported using an assistive device for mobility and support.

#### Preliminary Estimates of Change in Outcome Measures for Exploratory Aim

Table 4 presents changes in the outcome measures for the exploratory aim of the study. Compared to the control group, intervention group scores for the short-form 12 health survey physical component (*P*=.04) and FSQ basic ADL (*P*=.02) improved significantly, and BPI pain interference showed a trend of improvement (*P*=.07). There were no group differences on other measures. The effect size in the change of the outcome measures was small (Cohen *d* ranged from 0.2 to 0.4)

**Table 4:** Comparison of difference between the intervention (n=34) and control group (n=15) at the posttest^a^.

Outcome measure			Baseline, mean (SD)	Posttest, mean (SD)		Adjusted group difference (SE) at posttest^b^		95% CI for adjusted group difference		*P *value for adjusted group difference	Cohen *d* for effect size
**SF12^c^ physical component**											
	Intervention	45.6 (9.3)		48.5 (7.8)	4.0 (1.9)		0.1 to 7.8		.04		0.2
	Control	41.9 (11.7)		44.1 (9.8)							
**SF12 mental component**											
	Intervention	52.6 (10.5)		53.7 (8.8)	–0.5 (3.0)		–6.5 to 5.5		.89		0.3
	Control	56.4 (7.6)		56.9 (6.9)							
**FSQ^d^ basic activities of daily living^e^**											
	Intervention	-0.1 (0.9)		11.9 (0.3)	0.7 (0.3)		0.1 to 1.3		.02		N/A^f^
	Control	-0.01 (1.1)		11.2 (1.6)							
**FSQ intermediate activities of daily living^e^**											
	Intervention	n/a		19.9 (5.8)	1.3 (1.8)		–2.3 to 4.9		.47		N/A
	Control	n/a		17.5 (6.0)							
**BPI^g^ pain severity**											
	Intervention	2.5 (2.8)		1.5 (2.1)	0.3 (0.6)		–0.9 to 1.5		.58		0.0
	Control	2.4 (2.9)		1.8 (2.4)							
**BPI pain interference**											
	Intervention	1.8 (2.4)		0.9 (1.5)	–1.2 (0.6)		–2.5 to 0.1		.07		0.4
	Control	1.9 (2.5)		1.9 (2.8)							

^a^Sample sizes varied for different variables due to missing data.

^b^Adjusted difference in change scores from baseline to posttest between intervention and control group with adjustment to selected covariates.

^c^SF-12: short-form 12 health survey.

^d^FSQ: functional status questionnaire.

^e^Factor score of 5-time sit to stand, 50-foot fast walk, 6-minute walk, and forward reach was used to control the difference at baseline.

^f^N/A: not applicable.

^g^BPI: brief pain inventory.

## Discussion

### Principal Findings

*FITxOlder* is a community-based, mHealth-facilitated mind-body exercise program tailored to promote healthy aging among low-income older Latino adults with chronic health conditions. The findings indicate both the feasibility of participant recruitment and retention practices, as well as participants’ acceptance and satisfaction with the program. The participants in the intervention group also showed promising favorable responses in regard to measures of QoL and basic ADL.

*FITxOlder* showed acceptable feasibility of implementation, comparable to or better than published Qigong [55-57] and Tai Chi research [58-61]. An overwhelming majority of participants regularly attended the biweekly group sessions and achieved the weekly exercise goal. This high level of attendance and completion was maintained until COVID-19 hit the study community (weeks 5-8). The successful rate of reaching the study participants for the weekly phone calls (≥50%) was also acceptable considering the ongoing COVID-19 pandemic. We speculate that the acceptance of Five Animal Play is due to older Latinos being prone to seek alternative or nontraditional forms of health care or services partly due to experiences of disparate care and limited access to quality care [62,63].

*FITxOlder* participants reported positive exercise-induced feelings (eg, revitalization, tranquility, and positive engagement) associated with increased exercise efficacy and future exercise intentionality in older adults participating in low to moderate-intensity exercise programs [64,65]. The scores for physical exhaustion were slightly higher than those reported previously among young and middle-aged adults participating in low-impact aerobics or strength exercise programs and suggested that the Five Animal Play exercise program was equivalent to moderate levels of physical exertion in the older participants [65]. Based on both participant self-report and CHW documentation, importantly, there were no study-related adverse events. Our findings are consistent with other studies that have incorporated Qigong exercises and suggest the relative safety of Qigong practice for older adults with chronic health conditions [26,27]. Furthermore, previous research indicates that simplicity, low to moderate physical exertion demands, safety, and a sense of mastery motivate older adults to participate and continue PA [8,66].

Participants in both the intervention and control groups reported high levels of program satisfaction, which has been associated with high levels of program quality and patient services in medical care, mental health, and community settings [67,68]. Of note, the high level of program satisfaction among control participants indicated the appropriateness and acceptability of the placebo control treatment.

### Changes in Outcome Measures

We explored the effect of *FITxOlder* on physical and cognitive function and QoL as part of the feasibility study. Positive changes in the physical component of QoL, physical function, and pain interference were consistent with previous mind-body studies conducted in Western and non-Western older adults [17,26,27]. However, the size of the treatment effect was small. It is possible that the small effect size on physical health outcomes was related to the use of an attention placebo control group [69]. Another possibility is that the potential benefits of Qigong exercise were not fully realized due to the COVID reduction of in-person sessions from 12 weeks to 4 weeks which might have reduced the gain of confidence and proficiency in performing the Five Animal Play [70]. In a prior Qigong study, study participants were able to practice the routines at home after 4 weeks of in-person instruction but continued in-person sessions for the next 6 months [71]. On the other hand, we speculate that the lack of group differences on psychosocial outcomes was due to the weekly CHW phone calls to all participants (both treatment and control) who might have been experiencing social isolation and loneliness due to COVID-19, with the phone calls acting as an important source of attention and social support [72]. A longitudinal study found that individuals who experienced a greater sense of social connection and engagement with others reported a reduced level of pain severity but not pain interference [73].

### Comparison With Prior Work

Five Animal Play, one of the earliest forms of mind-body practice in traditional Chinese medicine, is widely practiced in China [40] yet has received limited attention in mind-body research conducted among Western populations [40,74]. Five Animal Play is relatively easy to scale and has much lower demands for space and equipment or instructor qualification and certification than Tai Chi or Yoga. The repetitive play-like routines of Five Animal Play contribute to reduced seriousness or “religiosity” and increases its appeal and acceptance in Western populations without undermining the therapeutic mechanisms of mind-body exercise commonly endorsed by CAM [74. Interventions that utilize simplified forms of Tai Chi, such as “Tai Chi: Moving for Better Balance”[75] and “Qigong/Tai Chi Easy” [71] consisting of repetitive routines of a small number of core Tai Chi or Qigong movements, have demonstrated efficacy and improvement in clinical indicators and QoL for older adults and those with neurodegenerative movement impairments or cancers (including cancer survivors) [44]. Dissemination studies of “Tai Chi: Moving for Better Balance” by lay Tai Chi instructors have demonstrated both acceptable feasibility and acceptability in community-based aging programs serving low-income and immigrant communities in the United States [76,77]. Similarly, we found that CHWs who received brief training could deliver the instruction of Five Animal Play to participants. Similar to previous studies [59,71], the use of study-produced videos allowed the participants to independently practice Five Animal Play from the beginning of the intervention. It was likely that the study videos served as a role model and reinforcement for the continuation of exercise in addition to the text reminders to meet the weekly exercise goal [42]. To our knowledge, this is the first study in which CHWs delivered a Qigong program. Areas for further research include an assessment of promoting participation in healthy aging programs that employ CHWs and incorporate mHealth facilitation.

### Strengths and Limitations

The strengths of *FITxOlder* include the CBPR approach and the use of a framework for the cultural adaptation of the evidence-based intervention [37]. The working group provided guidance and input in the adaptation of Five Animal Play and the delivery of the program. As a result, we were able to preemptively address issues and concerns regarding the biomechanics related to movement difficulty and safety (eg, speed, range, and exertion force), pedagogical approach (eg, instruction strategies, cues for breathing and movement, and instructor qualification), and cultural appropriateness (eg, music, animal symbolism, and culture-related analogies). Furthermore, we demonstrated that the program could be delivered by CHWs who have received a brief, focused training, in contrast to the high level of instructor qualifications and certification reported in other Qigong studies [26]. This is key since critiques of community interventions using Tai Chi or Yoga include the limited potential for scale and reproducibility based on high standards for instructor qualification and certification [18,78]. Finally, we used a “transcreation” approach [31] to guide the research team in evaluating scientific evidence and making design decisions. As a result, *FITxOlder* incorporated community-based strategies that fostered increased retention, attendance, and achievement of the exercise goal and increased the likelihood of sustainability. Future studies should compare the acceptability of Five Animal Play with low-impact aerobics, strength exercises, and other forms of meditative movement in older Latino adults and other vulnerable groups.

The *FITxOlder* intervention also benefited from facilitation by bilingual CHWs and mHealth tools (eg, preloaded Five Animal Play videos on a tablet, text messages, and telephone calls for instruction and support). CHWs can increase culturally appropriate delivery of information and support consistent with the participant’s beliefs and values and promote the participant’s engagement in community-based health promotion, even in the face of social distance restrictions imposed due to the COVID-19 pandemic [79]. Others also demonstrated that CHWs effectively implemented community-based exercise programs to improve mental health in older Latino adults [80]. The use of mHealth interventions to promote PA in older adults is an emerging field that has demonstrated promising short-term effects and the need for strong social support to engage the participants [30]. Our study findings also suggest that “live-person” social support from CHWs and facilitated by mHealth are critical to engage participants and maintain program participation [30].

The use of an active control was another strength that disentangled placebo effects from social support, attention, and expectancy in mind-body interventions, especially in older adults [69,81]. Some have questioned whether the effectiveness of CAM interventions is the result of a placebo effect due to the power of suggestion and attention experienced by the study participants [82]. We found similar levels of retention and satisfaction among both the intervention and control group participants, lending credence to the differences found in the study outcome measures [81].

Several limitations weakened the internal and external validity of our research. Due to COVID-19 epidemic restrictions, we were unable to assess all study outcomes with one standardized protocol at baseline and posttest. Given that posttest measures were conducted by phone rather than in person, study outcomes should be interpreted with caution. It is possible that posttest measurement by phone may have impacted the validity of the measures. Similarly, we could not examine potential differences between the 2 versions of Five Animal Play due to limited ability to conduct on-site observations after the first 4 weeks of intervention due to COVID-19. When participants transitioned to home practice, skill acquisition was in an early stage, and we were unable to ascertain the extent to which shortened in-person instruction may have impacted participants’ understanding of and ability to practice the movement routines as demonstrated in the study-produced videos.

In contrast, the reported direct instruction time was 12 weeks or longer in published Qigong studies [26,27]. Furthermore, we did not evaluate the level of proficiency of participants performing Five Animal Play at different stages of the learning progression to explore whether the level of skill proficiency was related to program attendance, satisfaction with the program, and changes in the outcome measures at the posttest [26]. For example, we were not able to assess the extent to which participants mastered the techniques of blending breathing with movement and mental quieting, which is essential to Qigong practice [74]. However, some studies found that Qigong interventions with and without a focus on teaching breathing and mental quieting had similar impacts on QoL, cognitive function, depression, and sleep quality in cancer survivors [24,83]. Finally, we did not explore the potential influence of Chinese cultural and Qigong-related beliefs on *FITxOlder* feasibility, acceptability, and outcomes. Of note, we purposefully avoided introducing the concept of “Qi” to participants due to the lack of an informed approach for mixing and matching Chinese and Latino culture-related health beliefs. Further research is needed to understand how culture-related Qigong beliefs influence the feasibility and health effects in non-Chinese participants.

### Conclusions

Findings from this research indicated the feasibility and acceptability of CHW-delivery of a traditional Chinese Qigong exercise with the facilitation of mHealth tailored for older low-income Latino adults in a community-based healthy aging program. However, the COVID-19 pandemic required a revision of the intervention protocol and prevented a full test of the feasibility of the *FITxOlder* program and participants’ responsiveness in study outcomes. Future research needs to replicate the study and compare the feasibility of Five Animal Play with other low-and-moderate intensity exercise programs with long-term follow-up.

## Appendix

Multimedia Appendix 1 Supplementary tables.

## Abbreviations

ADL: activities of daily living
BPI: brief pain inventory
CAM: complementary and alternative medicine
CBPR: community-based participatory research
CHW: community health worker
CSQ: client satisfaction questionnaire.
EBI: evidence-based interventions
EFI: exercise-induced feeling inventory
FITxOlder: Function Improvement Exercises for Older Sedentary Community-Dwelling Latino Residents
FSQ: functional status questionnaire
mHealth: mobile health
NIH: National Institutes of Health
PA: physical activity
QoL: quality of life
RCT: randomized controlled trials

## References

[1] The Adminstration for Community Living (2019). Profile of Older Americans.

[2] Brenner AB, Clarke PJ (2018). Understanding Socioenvironmental Contributors to Racial and Ethnic Disparities in Disability Among Older Americans. Res Aging.

[3] Christine B, Teague R, Melissa B (2017). Multiple Chronic Conditions in the United States.

[4] Ashman JJ, Beresovsky V (2013). Multiple chronic conditions among US adults who visited physician offices: data from the National Ambulatory Medical Care Survey, 2009. Prev Chronic Dis.

[5] Ward BW, Schiller JS (2013). Prevalence of multiple chronic conditions among US adults: estimates from the National Health Interview Survey, 2010. Prev Chronic Dis.

[6] The US Dept of Health and Human Service (2018). Physical activity guidelines for Americans, 2nd edition.

[7] Watson KB, Carlson SA, Gunn JP, Galuska DA, O'Connor A, Greenlund KJ, Fulton JE (2016). Physical Inactivity Among Adults Aged 50 Years and Older - United States, 2014. MMWR Morb Mortal Wkly Rep.

[8] Olanrewaju O, Kelly S, Cowan A, Brayne C, Lafortune L (2016). Physical Activity in Community Dwelling Older People: A Systematic Review of Reviews of Interventions and Context. PLoS One.

[9] Zubala A, MacGillivray S, Frost H, Kroll T, Skelton DA, Gavine A, Gray NM, Toma M, Morris J (2017). Promotion of physical activity interventions for community dwelling older adults: A systematic review of reviews. PLoS One.

[10] Sansano-Nadal O, Gine-Garriga M, Brach JS, Wert DM, Jerez-Roig J, Gueraa-Balic M, Oviedo G, Fortuno J, Gomara-Toldra N, Soto-Bagaria L, Perez LM, Inzitari M, Sola I, Martin-Borras C, Roche M (2019). Exercise-Based Interventions to Enhance Long-Term Sustainability of Physical Activity in Older Adults: A Systematic Review and Meta-Analysis of Randomized Clinical Trials. Int J Environ Res Public Health.

[11] National Center for Complementary and Alternative Medicine (2011). Exploring the Science of Complementary and Alternative Medicine.

[12] Sharp D, Lorenc A, Morris R, Feder G, Little P, Hollinghurst S, Mercer SW, MacPherson H (2018). Complementary medicine use, views, and experiences: a national survey in England. BJGP Open.

[13] Larkey L, Jahnke R, Etnier J, Gonzalez J (2009). Meditative movement as a category of exercise: implications for research. J Phys Act Health.

[14] Chen X, Cui J, Li R, Norton R, Park J, Kong J, Yeung A (2019). Dao Yin (a.k.a. Qigong): Origin, Development, Potential Mechanisms, and Clinical Applications. Evid Based Complement Alternat Med.

[15] Wang YT, Huang G, Duke G, Yang Y (2017). Tai Chi, Yoga, and Qigong as Mind-Body Exercises. Evidence-Based Complementary and Alternative Medicine.

[16] Horowitz S (2009). Evidence-Based Health Benefits of. Alternative and Complementary Therapies.

[17] Jahnke R, Larkey L, Rogers C, Etnier J, Lin F (2010). A comprehensive review of health benefits of qigong and tai chi. Am J Health Promot.

[18] Klein PJ, Baumgarden J, Schneider R (2019). Qigong and Tai Chi as Therapeutic Exercise: Survey of Systematic Reviews and Meta-Analyses Addressing Physical Health Conditions. Altern Ther Health Med.

[19] Zhang Y, Hu R, Han M, Lai B, Liang S, Chen B, Robinson N, Chen K (2020). Evidence Base of Clinical Studies on Qi Gong: A Bibliometric Analysis. Complementary Therapies in Medicine.

[20] Kelley GA, Kelley KS (2015). Meditative Movement Therapies and Health-Related Quality-of-Life in Adults: A Systematic Review of Meta-Analyses. PLoS One.

[21] Morgan N, Irwin MR, Chung M, Wang C (2014). The effects of mind-body therapies on the immune system: meta-analysis. PLoS One.

[22] Oh B, Bae K, Lamoury G, Eade T, Boyle F, Corless B, Clarke S, Yeung A, Rosenthal D, Schapira L, Back M (2020). The Effects of Tai Chi and Qigong on Immune Responses: A Systematic Review and Meta-Analysis. Medicines (Basel).

[23] Morone NE, Greco CM (2007). Mind–Body Interventions for Chronic Pain in Older Adults: A Structured Review: Table 1. Pain Med.

[24] Larkey LK, Roe DJ, Weihs KL, Jahnke R, Lopez AM, Rogers CE, Oh B, Guillen-Rodriguez J (2015). Randomized controlled trial of Qigong/Tai Chi Easy on cancer-related fatigue in breast cancer survivors. Ann Behav Med.

[25] Jahnke R, Larkey L, Rogers C (2010). Dissemination and benefits of a replicable Tai Chi and Qigong program for older adults. Geriatr Nurs.

[26] Kemp CA (2004). Qigong as a therapeutic intervention with older adults. J Holist Nurs.

[27] Dong X, Bergren S (2016). Qigong among older adults: a global review. Clin Res Trial.

[28] Hansen-Kyle L (2005). A concept analysis of healthy aging. Nurs Forum.

[29] Depp CA, Glatt SJ, Jeste DV (2007). Recent advances in research on successful or healthy aging. Curr Psychiatry Rep.

[30] Jonkman NH, van Schooten KS, Maier AB, Pijnappels M (2018). eHealth interventions to promote objectively measured physical activity in community-dwelling older people. Maturitas.

[31] Nápoles AM, Stewart AL (2018). Transcreation: an implementation science framework for community-engaged behavioral interventions to reduce health disparities. BMC Health Serv Res.

[32] Pasick RJ, D'onofrio CN, Otero-Sabogal R (2016). Similarities and Differences Across Cultures: Questions to Inform a Third Generation for Health Promotion Research. Health Education Quarterly.

[33] (2019). Stage model for behavioral intervention development. National Institutes of Health and National Institute on Aging.

[34] Onken LS, Carroll KM, Shoham V, Cuthbert BN, Riddle M (2014). Reenvisioning Clinical Science: Unifying the Discipline to Improve the Public Health. Clin Psychol Sci.

[35] Yin Z, Li S, Land WM, Ullevig SL, Juarez F, Hernández AE, Ortega C, Patel NK, Simmonds MJ (2021). Higher levels of physical activity buffered the negative effect of pain severity on physical frailty in older Latinx adults. Geriatr Nurs.

[36] Sofolahan-Oladeinde Y, Mullins CD, Baquet CR (2015). Using community-based participatory research in patient-centered outcomes research to address health disparities in under-represented communities. J Comp Eff Res.

[37] Center for Community Health Development (1994). Chapter 19 Choosing and Adapting Community Interventions.

[38] Chinese Health Qigong Association (2007). Chinese Health Qigong: Wu Qin Xi.

[39] Chinese Health Qigong Association (2010). Chinese Health Qigong: Ba Duan Jin.

[40] Balaneskovic S (2018). Hua Tuo's Wu Qin Xi (Five Animal Frolics) movements and the logic behind it. Chin Med Cult.

[41] Romero M, Hondagneu-Sotelo P, Ortiz V (1997). Challenging fronteras: Structuring Latina and Latino lives in the U.S. An anthology of readings.

[42] Lee L, Arthur A, Avis M (2008). Using self-efficacy theory to develop interventions that help older people overcome psychological barriers to physical activity: a discussion paper. Int J Nurs Stud.

[43] Dorcas A, Yung P (2003). Qigong: Harmonising the breath, the body and the mind. Complementary Therapies in Nursing and Midwifery.

[44] Larkey L, Jahnke R, Etnier J, Gonzalez J (2009). Meditative movement as a category of exercise: implications for research. J Phys Act Health.

[45] National Council on Aging Aging Mastery Program® Research and Evidence-Base.

[46] Lise G, Rejeski W (1993). The Exercise-Induced Feeling Inventory: Development and Initial Validation. Journal of Sport and Exercise Psychology.

[47] Attkisson CC, Greenfield TK (1994). Client Satisfaction Questionnaire-8Service Satisfaction Scale-30. The use of psychological testing for treatment planning outcome assessment.

[48] Calder PC, Carding SR, Christopher G, Kuh D, Langley-Evans SC, McNulty H (2018). A holistic approach to healthy ageing: how can people live longer, healthier lives?. J Hum Nutr Diet.

[49] Jette AM, Davies AR, Cleary PD, Calkins DR, Rubenstein LV, Fink A, Kosecoff J, Young RT, Brook RH, Delbanco TL (1986). The Functional Status Questionnaire: reliability and validity when used in primary care. J Gen Intern Med.

[50] Smith A (1982). Symbol digit modalities test: Manual.

[51] Simmonds MJ, Olson SL, Jones S, Hussein T, Lee CE, Novy D, Radwan H (1998). Psychometric characteristics and clinical usefulness of physical performance tests in patients with low back pain. Spine (Phila Pa 1976).

[52] Ware JJ, Kosinski M, Turner-Bowker D, Sundaram M, Gandek BM (2009). User's manual for the SF-12v2 Health Survey 2nd ed.

[53] Poquet N, Lin C (2016). The Brief Pain Inventory (BPI). J Physiother.

[54] Bohlmeijer E, ten Klooster PM, Fledderus M, Veehof M, Baer R (2011). Psychometric properties of the five facet mindfulness questionnaire in depressed adults and development of a short form. Assessment.

[55] Myers JS, Mitchell M, Krigel S, Steinhoff A, Boyce-White A, Van Goethem K, Valla M, Dai J, He J, Liu W, Sereika SM, Bender CM (2019). Qigong intervention for breast cancer survivors with complaints of decreased cognitive function. Support Care Cancer.

[56] Oh B, Butow P, Mullan B, Clarke S, Beale P, Pavlakis N, Kothe E, Lam L, Rosenthal D (2010). Impact of medical Qigong on quality of life, fatigue, mood and inflammation in cancer patients: a randomized controlled trial. Ann Oncol.

[57] Sarmento CVM, Moon S, Pfeifer T, Smirnova IV, Colgrove Y, Lai SM, Liu W (2020). The therapeutic efficacy of Qigong exercise on the main symptoms of fibromyalgia: A pilot randomized clinical trial. Integr Med Res.

[58] Logghe I, Verhagen A, Rademaker A, Zeeuwe P, Bierma-Zeinstra S, Van Rossum E, Faber M, Van Haastregt JCM, Koes B (2011). Explaining the ineffectiveness of a Tai Chi fall prevention training for community-living older people: a process evaluation alongside a randomized clinical trial (RCT). Arch Gerontol Geriatr.

[59] Katrancha ED, Hoffman LA, Zullo TG, Tuite PK, Garand L (2015). Effects of a video guided T'ai Chi group intervention on center of balance and falls efficacy: a pilot study. Geriatr Nurs.

[60] Fong SSM, Ng SSM, Lee HW, Pang MYC, Luk WS, Chung JWY, Wong JYH, Masters RSW (2015). The effects of a 6-month Tai Chi Qigong training program on temporomandibular, cervical, and shoulder joint mobility and sleep problems in nasopharyngeal cancer survivors. Integr Cancer Ther.

[61] Day L, Hill K, Stathakis V, Flicker L, Segal L, Cicuttini F, Jolley D (2015). Impact of tai-chi on falls among preclinically disabled older people. A randomized controlled trial. J Am Med Dir Assoc.

[62] Ortiz BI, Shields KM, Clauson KA, Clay PG (2007). Complementary and alternative medicine use among Hispanics in the United States. Ann Pharmacother.

[63] Cherniack EP, Ceron-Fuentes J, Florez H, Sandals L, Rodriguez O, Palacios JC (2008). Influence of race and ethnicity on alternative medicine as a self-treatment preference for common medical conditions in a population of multi-ethnic urban elderly. Complement Ther Clin Pract.

[64] Rhodes RE, Kates A (2015). Can the Affective Response to Exercise Predict Future Motives and Physical Activity Behavior? A Systematic Review of Published Evidence. Ann Behav Med.

[65] Barnett F (2013). The effect of exercise on affective and self-efficacy responses in older and younger women. J Phys Act Health.

[66] Schutzer K, Graves B (2004). Barriers and motivations to exercise in older adults. Prev Med.

[67] Schulte SJ, Meier PS, Stirling J (2011). Dual diagnosis clients' treatment satisfaction - a systematic review. BMC Psychiatry.

[68] Attkisson C, Greenfield T, Maruish ME (2004). The UCSF Client Satisfaction Scales: I. The Client Satisfaction Questionnaire-8. The use of psychological testing for treatment planning outcomes assessment: Instruments for adults, Volume 3, 3rd ed.

[69] Lindquist R, Wyman JF, Talley KMC, Findorff MJ, Gross CR (2007). Design of control-group conditions in clinical trials of behavioral interventions. J Nurs Scholarsh.

[70] Rogers CE, Larkey LK, Keller C (2009). A review of clinical trials of tai chi and qigong in older adults. West J Nurs Res.

[71] Larkey LK, Roe DJ, Smith L, Millstine D (2016). Exploratory outcome assessment of Qigong/Tai Chi Easy on breast cancer survivors. Complement Ther Med.

[72] Cudjoe TKM, Kotwal AA (2020). "Social Distancing" Amid a Crisis in Social Isolation and Loneliness. J Am Geriatr Soc.

[73] Karayannis N, Baumann I, Sturgeon J, Melloh M, Mackey S (2019). The Impact of Social Isolation on Pain Interference: A Longitudinal Study. Ann Behav Med.

[74] Guo Y, Xu M, Wei Z, Hu Q, Chen Y, Yan J, Wei Y (2018). Beneficial Effects of in the Improvement of Health Condition, Prevention, and Treatment of Chronic Diseases: Evidence from a Systematic Review. Evid Based Complement Alternat Med.

[75] Li F (2014). Transforming traditional Tai Ji Quan techniques into integrative movement therapy-. J Sport Health Sci.

[76] Fink D, Houston K (2014). Implementing an evidence-based Tai Ji Quan program in a multicultural setting: A pilot dissemination project. Journal of Sport and Health Science.

[77] Leung J (2014). Implementing Tai Ji Quan: Moving for Better Balance in real-world settings: Success and challenges. Journal of Sport and Health Science.

[78] Harmer P (2014). So much research, so little application: Barriers to dissemination and practical implementation of Tai Ji Quan. J Sport Health Sci.

[79] Cherrington A, Ayala GX, Elder JP, Arredondo EM, Fouad M, Scarinci I (2010). Recognizing the diverse roles of community health workers in the elimination of health disparities: from paid staff to volunteers. Ethn Dis.

[80] Mays AM, Kim S, Rosales K, Au T, Rosen S (2021). The Leveraging Exercise to Age in Place (LEAP) Study: Engaging Older Adults in Community-Based Exercise Classes to Impact Loneliness and Social Isolation. Am J Geriatr Psychiatry.

[81] Gonçalves Mário, Matos LC, Duarte L, Machado J, Greten HJ, Franconi G (2020). Problems of scientific methodology related to placebo control in Qigong studies: A systematic review. J Bodyw Mov Ther.

[82] Park CM (2002). Diversity, the individual, and proof of efficacy: complementary and alternative medicine in medical education. Am J Public Health.

[83] Larkey L, Huberty J, Pedersen M, Weihs K (2016). Qigong/Tai Chi Easy for fatigue in breast cancer survivors: Rationale and design of a randomized clinical trial. Contemp Clin Trials.

