# Supplementary material for: Adapting Chinese Qigong Mind-Body Exercise for Healthy Aging in Older Community-Dwelling Low-income Latino Adults: Pilot Feasibility Study
Source: JMIR Aging. 2021 Nov 1;4(4):e29188. doi: 10.2196/29188 (PMC8593812; doi:10.2196/29188)
Supplement: Multimedia Appendix 1 [file aging_v4i4e29188_app1.docx]

Supplement Table 1. Summary of State-of-Art Review

| 1. A majority of early randomized trials (RCTs) were conducted in China including Hong Kong and Taiwan that tended to have favorable results than the studies conducted in Western populations. [1-5] |
| --- |
| 1. Study participants represented a broad spectrum of populations of various health condition and sociocultural background, e.g., healthy college students, healthy adults, older adults, cancer survivors, (older) adults with chronic disease. [1-7] |
| 1. Research is spars in populations identified as low-income or racial/ethnic minority. [3, 7, 8] |
| 1. Tai Chi or modified Tai Chi is the most often used exercise followed by Eight Pieces of Brocade and Five Animal Play.[4, 9-20] |
| 1. Qigong is a safe practice with minimum medical supervision for all population groups including older adults and individuals with chronic health conditions.[1-4, 21] |
| 1. There is an emerging body of evidence supporting the cost-effectiveness and cost-saving as a therapeutic form of complementary and alternative medicine. [2-5, 22] |
| 1. A majority of the studies were short-term interventions ranging 3 to 6 months without long-term follow-up.[1, 3, 9, 23-25] |
| 1. Few studies examined culture or social aspects of Qigong ant its influence in study outcomes.[13, 25, 26] |
| 1. Intervention settings were mainly group or home-based.[3, 7, 13, 19, 26, 27] |
| 1. Study outcomes were assessed using self-administrated questionnaires on sleep, depression, quality of life, pain, and stress; physical and cognitive function with clinical assessment tools; physical fitness; management of chronic diseases (diabetes, cancer survivorship, hypertension) and conditions (falls, sleep, depression); blood assay of cardiometabolic risk and inflammatory responses; and electronic imaging. [1-7, 24, 25, 28-34] |
| 1. Most studies used no treatment, usual care, or health education as control treatment that mainly refrained the participants from physical activity.[1-7, 13, 19, 26, 27] [35, 36] |
| 1. Compared to Tai Chi and Yoga, Five Animal Play and Eight Pieces of Brocade are easier to learn and sustain due the repeated nature of the exercise, low to moderate level of physical exertion, and faster learning curve. [5, 9, 37, 38] |
| 1. There is no standardized objective method to judge or evaluate the level of mastery (level of skill or quality) or quantify or measure the amount of volume or “dose” of the exercise.[5, 7, 13, 25, 39, 40] |
| 1. Most participants learned Qigong and Yoga under the instruction of professional practitioners or highly trained instructors. As a result, there is a lack of details in Qigong exercises or instruction program used in the interventions. [7, 10, 25, 41, 42] |
| 1. Quality of Qigong research is weakened by methodological issues, such as lack of clarity of study design, small sample size, short-study duration, lack of long-term follow-up, lack of description of Qigong exercise in the study, and inadequate report of feasibility, such as, limited report of fidelity of implementation, mixed report of participant retention, attendance and acceptance. .[1-7, 13, 19, 26, 27] [35, 36] |
| 1. Future research questions:  - What are the underlying mechanisms of Qigong (low to moderate intensity and controlled breathing) to influence health outcomes compared to the regular exercise intervention with known mechanisms? - How does Qigong influence different dimensions of physical and cognitive function compared to regular exercise (aerobic and/or strengthening) program? - Does the effect of Qigong intervention vary in participants who have different level of mastery or proficiency of Qigong (accuracy in performing the movements, use of controlled breathing, level of mental focus)? - If, why, or how is Qigong more enjoyable and acceptable compared to regular exercise program to improve compliance and motivation in home-based intervention? - What are the challenges/Issues to implement Qigong programs in non-Chinese populations? - To what extend is it important to introduce the cultural aspects of Qigong to the study participants? - To what extend does the role of Chinese culture or belief in Qigong practice influence the study outcomes? - How to control the placebo effect in studies examining the effects of Qigong exercise? |

Note: The reviews were conducted based on search of published literature in English language that examined Qigong exercises, such as Tai Chi, Five Animal Play and Eight Pieces of Brocade in systematic reviews and expert reviews

Supplement Table 2. Group Session Layout (60 minutes)

| Activity | Approximate Duration |
| --- | --- |
| Meet-and-greet (Repeated during all sessions)   - Attendance - Sharing of experience from previous week | 5-10 minutes |
| Warm-up (Repeated during all sessions)   - Stretching - Abdominal breathing - Basic movements of Five Animal Play | 5 minutes |
| Instructor-led practice of Five Animal Play following video | 13 minutes |
| Instruction of Five Animal Play   - Opening routine - Routine 1: Tiger (subroutine 1, Raising tiger paws; subroutine 2, Seizing the prey - Routine 2: Deer (subroutine 1, Colliding with the antlers; subroutine 2, Running like a deer) - Routine 3: Monkey (subroutine 1, Lifting the monkey’s paws; subroutine 2, Picking fruits) - Routine 4: Bear (subroutine 1, Rotating the waist like a bear; subroutine 2, Swaying like a bear - Routine 5: Crane (subroutine 1, Stretching upward; subroutine 2, Flying like a bird - Closing routine | 10 minutes |
| Instructor-led practice of Five Animal Play following video | 13 minutes |
| Closing remarks   - Complete exercise feeling survey and exercise log - Reinforce home exercise goals - Problem solving (movement and technical related) | 5-10 minutes |

Supplement Table 3. Health History of Study Participants at Baseline

|  | All  (n=49) | Intervention  (n=34) | Control  (n=15) |
| --- | --- | --- | --- |
| Has a doctor ever said your blood pressure was too high?  Yes (n, %) | 28, 57.1% | 20, 58.% | 8, 53.3% |
| Do you ever have pain in your chest or heart?  Yes (n, %) | 8, 16.3% | 4, 11.8% | 4, 26.7% |
| Has a doctor ever said that you have or have had heart trouble, an abnormal electrocardiogram (ECG or EKG), heart attack or coronary?)  Yes (n, %) | 10, 20.4% | 5, 14.7% | 5, 33.3% |
| Do you now have or have you recently experienced leg cramps?  Yes (n, %) | 14, 28.6% | 9, 26.5% | 5, 33.3% |
| Has a doctor ever told you your cholesterol level was high? | 30, 61.2% | 18, 52.9% | 12, 80.0% |
| Do you now have or have you recently experienced chronic cough | 5, 10.2% | 1, 2.9% | 4, 26.7% |
| Do you now have or have you recently experienced increased anxiety and depression | 14, 28.6% | 8, 23.5% | 6, 40.0% |
| Do you now have or have you recently experienced depression symptoms | 10, 20.4% | 8, 23.5% | 2, 13.3% |
| Do you now have or have you recently experienced Migraine or recurrent headache | 5, 10.2% | 4, 11.8% | 1, 6.7% |
| Do you now have or have you recently experienced stomach problems | 12, 24.5% | 7, 20.6% | 5, 33.3% |
| Do you now have or have you recently experienced vision/hearing problem | 13, 26.5% | 6, 17.6% | 7, 46.7% |
| Do you now have or have you recently experienced Glaucoma | 7, 14.3% | 3, 8.8% | 4, 26.7% |
| Do you now have or have you recently experienced unexplained weight loss | 1, 2.0% | 1, 2.9% | 0, 0.0% |
| Do you now have or have you recently experienced Cataract | 16, 32.7% | 11, 32.4% | 5, 33.3% |
| Do you now have or have you recently experienced urine loss | 11, 22.4% | 7, 20.6% | 4, 26.7% |
| Do you now have or have you recently experienced symptom of heart problem?  0  1  2  3 or more | 39, 79.6%  7, 14.3%  1, 2.0%  2, 4.0% | 29, 85.3%  4, 11.8%  0, 0.0%  1, 2.9% | 10, 66.7%  3, 20.0%  1, 6.7%  1, 6.7% |
| Do you now have or have you recently experienced breathing problems?  0  1  2 or more | 43, 87.8%  3, 6.1%  3, 6.1% | 31, 91.2%  2, 5.9%  1, 2.9% | 12, 80.0%  1, 6.7%  2, 13.4% |
| Do you now have or have you recently experienced one or more symptoms of pain in lower extremity (foot, knee, ankle, leg, back)?  0  1  2 ore more | 21, 42.9%  11, 22.4%  17, 34.7% | 16, 47.1%  8, 23.5%  10, 29.4% | 5, 33.3%  3, 20.0%  7, 46.7% |
| Number of chronic health conditions (high blood pressure, heart trouble, increased anxiety/depression, stomach problem, and vision/hearing problem) the participants now have or have recently experienced  0  2  2 or more | 11, 22.4%  14, 28.6%  24, 49.0% | 9, 26.5%  13, 11.8%  12, 25.3% | 2, 13.3%  1, 6.7%  12, 67% |

**References**

1. Zhang Y-P, Hu R-X, Han M, Lai B-Y, Liang S-B, Chen B-J, et al. Evidence Base of Clinical Studies on Qi Gong: A Bibliometric Analysis. Complementary Therapies in Medicine. 2020 2020/05/01/;50:102392. doi: <https://doi.org/10.1016/j.ctim.2020.102392>.

2. Lee MS, Ernst E. Systematic reviews of t'ai chi: an overview. J Br J Sports Med. 2012;46(10):713-8.

3. Klein PJ, Baumgarden J, Schneider R. Qigong and Tai Chi as Therapeutic Exercise: Survey of Systematic Reviews and Meta-Analyses Addressing Physical Health Conditions. Alternative therapies in health and medicine. 2019;25(5):48-53. PMID: 31221939.

4. Kemp CA. Qigong as a Therapeutic Intervention With Older Adults. Journal of Holistic Nursing. 2004 2004/12/01;22(4):351-73. doi: 10.1177/0898010104269313.

5. Jahnke R, Larkey L, Rogers C, Etnier J, Lin F. A comprehensive review of health benefits of qigong and tai chi. Am J Health Promot. 2010 Jul-Aug;24(6):e1-e25. PMID: 20594090. doi: 10.4278/ajhp.081013-LIT-248.

6. Rogers C, Keller C, Larkey LK. Perceived Benefits of Meditative Movement in Older Adults. Geriatric Nursing. 2010 2010/01/01/;31(1):37-51. doi: <https://doi.org/10.1016/j.gerinurse.2009.10.002>.

7. Dong X, Bergren S, editors. Qigong among older adults: a global review. 2016.

8. Sharp D, Lorenc A, Morris R, Feder G, Little P, Hollinghurst S, et al. Complementary medicine use, views, and experiences: a national survey in England. BJGP Open. 2018 Dec;2(4):bjgpopen18X101614. PMID: 30723800. doi: 10.3399/bjgpopen18X101614.

9. Wang YT, Huang G, Duke G, Yang Y. Tai Chi, Yoga, and Qigong as Mind-Body Exercises. Evidence-Based Complementary and Alternative Medicine. 2017 2017/01/05;2017:8763915. doi: 10.1155/2017/8763915.

10. Stevens JA, Voukelatos A, Ehrenreich H. Preventing falls with Tai Ji Quan: A public health perspective. Journal of Sport and Health Science. 2014 2014/03/01/;3(1):21-6. doi: <https://doi.org/10.1016/j.jshs.2013.10.002>.

11. Song R, Grabowska W, Park M, Osypiuk K, Vergara-Diaz G, Bonato P, et al. The impact of Tai Chi and Qigong mind-body exercises on motor and non-motor function and quality of life in Parkinson's disease: A systematic review and meta-analysis. J Parkinsonism Related Disorders. 2017;41:3-13.

12. Sansano-Nadal O, Giné-Garriga M, Brach JS, Wert DM, Jerez-Roig J, Guerra-Balic M, et al. Exercise-Based Interventions to Enhance Long-Term Sustainability of Physical Activity in Older Adults: A Systematic Review and Meta-Analysis of Randomized Clinical Trials. Int J Environ Res Public Health. 2019 Jul 15;16(14). PMID: 31311165. doi: 10.3390/ijerph16142527.

13. Rogers CE, Larkey LK, Keller C. A review of clinical trials of tai chi and qigong in older adults. West J Nurs Res. 2009 Mar;31(2):245-79. PMID: 19179544. doi: 10.1177/0193945908327529.

14. Leung J. Implementing Tai Ji Quan: Moving for Better Balance in real-world settings: Success and challenges. Journal of Sport and Health Science. 2014 2014/03/01/;3(1):34-5. doi: <https://doi.org/10.1016/j.jshs.2013.12.001>.

15. Oh B, Butow P, Mullan B, Clarke S, Beale P, Pavlakis N, et al. Impact of medical Qigong on quality of life, fatigue, mood and inflammation in cancer patients: a randomized controlled trial. Ann Oncol. 2010 Mar;21(3):608-14. PMID: 19880433. doi: 10.1093/annonc/mdp479.

16. Klein P, Picard G, Schneider R, Oh B. International Expert Panel Consensus Guidelines for Structure and Delivery of Qigong Exercise for Cancer Care Programming. Medicines. 2017;4(3):54. PMID: PMC5622389. doi: 10.3390/medicines4030054.

17. Chinese Health Qigong Association. Chinese Health Qigong: Wu Qin Xi. Beijing, China: Chinese Health Qigong Association; Foreign Language Press2007.

18. Balaneskovic S. Hua Tuo's Wu Qin Xi (Five Animal Frolics) movements and the logic behind it. Chinese Medicine and Culture. 2018 October 1, 2018;1(3):127-34. doi: 10.4103/cmac.Cmac_32_18.

19. Antonishen K. Exercise mode heterogeneity among reported studies of the qigong practice Baduanjin. Journal of Bodywork and Movement Therapies. 2015 2015/04/01/;19(2):278-83. doi: <https://doi.org/10.1016/j.jbmt.2014.05.013>.

20. Trends in Yoga, Tai Chi, and Qigong Use: Differentiations Between Practices and the Need for Dialogue and Diffusion. American Journal of Public Health. 2019 05//;109(5):662-3. PMID: 135849136. doi: 10.2105/AJPH.2019.305042.

21. Han A, Judd M, Welch V, Wu T, Tugwell P, Wells GA. Tai chi for treating rheumatoid arthritis. J Cochrane Database of Systematic Reviews. 2004 (3).

22. Willison KD, Andrews GJ. Complementary medicine and older people: past research and future directions. Complement Ther Nurs Midwifery. 2004 May;10(2):80-91. PMID: 15135760. doi: 10.1016/s1353-6117(03)00106-9.

23. Kelley GA, Kelley KS. Meditative Movement Therapies and Health-Related Quality-of-Life in Adults: A Systematic Review of Meta-Analyses. PLoS One. 2015;10(6):e0129181. PMID: 26053053. doi: 10.1371/journal.pone.0129181.

24. Chen X, Cui J, Li R, Norton R, Park J, Kong J, et al. Dao Yin (a.k.a. Qigong): Origin, Development, Potential Mechanisms, and Clinical Applications. Evid Based Complement Alternat Med. 2019;2019:3705120-. PMID: 31772593. doi: 10.1155/2019/3705120.

25. Guo Y, Xu M, Wei Z, Hu Q, Chen Y, Yan J, et al. Beneficial Effects of Qigong Wuqinxi in the Improvement of Health Condition, Prevention, and Treatment of Chronic Diseases: Evidence from a Systematic Review. Evid Based Complement Alternat Med. 2018;2018:3235950. PMID: 30473716. doi: 10.1155/2018/3235950.

26. Vergeer I. Trends in Yoga, Tai Chi, and Qigong Use: Differentiations Between Practices and the Need for Dialogue and Diffusion. Am J Public Health. 2019 May;109(5):662-3. PMID: 30969825. doi: 10.2105/ajph.2019.305042.

27. Jiménez-Martín PJ. Tai Chi Chuan and Qigong in scientific research: present and future. / [Tai Chi Chuan y Qigong en el ámbito científico: presente y futuro. RICYDE Revista Internacional de Ciencias del Deporte. 2014;10(36):91-4. PMID: 95439302.

28. Klein PJ, Schneider R, Rhoads CJ. Qigong in cancer care: a systematic review and construct analysis of effective Qigong therapy. Support Care Cancer. 2016 Jul;24(7):3209-22. PMID: 27044279. doi: 10.1007/s00520-016-3201-7.

29. Klein P. Qigong in Cancer Care: Theory, Evidence-Base, and Practice. Medicines (Basel). 2017;4(1):2. PMID: 28930219. doi: 10.3390/medicines4010002.

30. Henz D, Schöllhorn WI. EEG Brain Activity in Dynamic Health Qigong Training: Same Effects for Mental Practice and Physical Training? Frontiers in Psychology. 2017 2017-February-07;8(154). doi: 10.3389/fpsyg.2017.00154.

31. Fang W, Man JKM, Lee E-KO, Taixiang W, Benson H, Fricchione GL, et al. The Effects of Qigong on Anxiety, Depression, and Psychological Well-Being: A Systematic Review and Meta-Analysis. Evidence-based Complementary & Alternative Medicine (eCAM). 2013;2013:1-16. PMID: 95496194. doi: 10.1155/2013/152738.

32. Chan ES, Koh D, Teo YC, Hj Tamin R, Lim A, Fredericks S. Biochemical and psychometric evaluation of Self-Healing Qigong as a stress reduction tool among first year nursing and midwifery students. Complementary Therapies in Clinical Practice. 2013 2013/11/01/;19(4):179-83. doi: <https://doi.org/10.1016/j.ctcp.2013.08.001>.

33. Oh B, Bae K, Lamoury G, Eade T, Boyle F, Corless B, et al. The Effects of Tai Chi and Qigong on Immune Responses: A Systematic Review and Meta-Analysis. Medicines (Basel). 2020 Jun 30;7(7). PMID: 32629903. doi: 10.3390/medicines7070039.

34. Ng BHP, Tsang HWH. Psychophysiological outcomes of health qigong for chronic conditions: A systematic review. Psychophysiology. 2009 03//;46(2):257-69. PMID: 36460419. doi: 10.1111/j.1469-8986.2008.00763.x.

35. Lan C, Chou S-W, Chen S-Y, Lai J-S, Wong M-K. The aerobic capacity and ventilatory efficiency during exercise in Qigong and Tai Chi Chuan practitioners. The American Journal Of Chinese Medicine. 2004;32(1):141-50. PMID: 15154293.

36. Chen K. Methodological challenges and research design in research study of qigong therapies. 2015. p. 228-48.

37. Jahnke RA, Larkey LK, Rogers C. Dissemination and Benefits of a Replicable Tai Chi and Qigong Program for Older Adults. Geriatric Nursing. 2010 2010/07/01/;31(4):272-80. doi: <https://doi.org/10.1016/j.gerinurse.2010.04.012>.

38. Larkey L, Jahnke R, Etnier J, Gonzalez J. Meditative movement as a category of exercise: implications for research. J Phys Act Health. 2009 Mar;6(2):230-8. PMID: 19420401. doi: 10.1123/jpah.6.2.230.

39. Jiménez-Martín PJ, Liu H, Melendez-Ortega A. How to study the relationship between Tai Chi Chuan, Qigong and medicine – A review of research frameworks. European Journal of Integrative Medicine. 2016 2016/12/01/;8(6):888-93. doi: <https://doi.org/10.1016/j.eujim.2016.11.012>.

40. Horowitz S. Evidence-Based Health Benefits of Qigong. Alternative and Complementary Therapies. 2009 2009/08/01;15(4):178-83. doi: 10.1089/act.2009.15401.

41. Harmer PA. So much research, so little application: Barriers to dissemination and practical implementation of Tai Ji Quan. Journal of Sport and Health Science. 2014 2014/03/01/;3(1):16-20. doi: <https://doi.org/10.1016/j.jshs.2013.10.005>.

42. Ng BHP, Tsang HWH, Ng BFL, So C-t. Traditional Chinese Exercises for Pulmonary Rehabilitation: EVIDENCE FROM A SYSTEMATIC REVIEW. Journal of Cardiopulmonary Rehabilitation and Prevention 2014;34(6):367-77. PMID: 01273116-201411000-00001. doi: 10.1097/hcr.0000000000000062.
